# Women’s empowerment and childhood malaria in sub-Saharan Africa: evidence from DHS, 2013–2022

**DOI:** 10.7189/jogh.16.04369

**Published:** 2026-10-23

**Authors:** Zhongfei Pei, Xin Ru, Xiaoyi Yu, Shuduo Zhou, Sok King Ong, Maha El Rabbat, Ming Xu

**Affiliations:** 1 Department of Global Health, School of Public Health, Peking University, Beijing, China; 2 Institute for Global Health and Development, Peking University, Beijing, China; 3 Institute of Food and Nutrition Development, Ministry of Agriculture and Rural Affairs, Beijing, China; 4 Peking University First Hospital, Xicheng District, Beijing, China; 5 PAPRSB Institute of Health Sciences, Universiti Brunei Darussalam, Jln Tungku Link, Bandar Seri Begawan, Brunei Darussalam; 6 Public Health Department, Faculty of Medicine, Cairo University, Egypt

**Keywords:** malaria, women’s empowerment, attitude to violence, social independence, decision-making, sub-Saharan Africa

## Abstract

**Background:**

Women’s empowerment is known to be associated with better access to child healthcare and household prevention practices. Children under five years of age depend on caregivers for access to malaria prevention, diagnosis, and treatment, making maternal agency potentially relevant to childhood malaria. However, evidence linking its multidimensional components to childhood malaria remains limited. We examined the association between women’s empowerment and childhood malaria among children in sub-Saharan Africa and explored whether these associations varied across countries and according to the children’s bed net utilisation.

**Methods:**

We conducted a cross-sectional secondary analysis of Demographic and Health Survey (DHS) data from 19 sub-Saharan African countries surveyed between 2013 and 2022. Weighted generalised linear mixed models with a primary sampling unit random intercept and country fixed effects examined associations between three Survey-based Women’s Empowerment Global index domains (attitude to violence, social independence and decision-making) and malaria test positivity among children under five years of age. Then, we ran country-specific analyses to assess between-country heterogeneity and exploratory adjustment, interaction, and stratified analyses to examine whether associations varied by the children’s bed net utilisation on the previous night.

**Results:**

Our sample comprised 79,739 children. Compared with low empowerment, medium (adjusted odds ratio (aOR) = 0.85; 95% confidence interval (CI) = 0.78–0.92), and high (aOR = 0.85; 95% CI = 0.80–0.90) empowerment within the attitude to violence domain were associated with lower odds of malaria positivity. Inverse associations were also observed for high social independence (aOR = 0.79; 95% CI = 0.74–0.84) and high decision-making empowerment (aOR = 0.89; 95% CI = 0.80–0.99), yet the medium levels of these domains were not clearly associated with malaria positivity. Between-country heterogeneity was identified for social independence and decision-making. Exploratory analyses suggested variation by bed net utilisation for attitude to violence and decision-making, but not social independence.

**Conclusions:**

Selected dimensions of women’s empowerment were associated with lower odds of childhood malaria, with the clearest association observed for high social independence. Variation across domains, countries, and household prevention contexts supports gender-sensitive malaria research and programme planning. Longitudinal and mixed-methods studies are needed to clarify the temporality of these associations and the underlying mechanisms.

Malaria continues to pose a major burden of infectious disease worldwide. According to the World Malaria Report 2025, an estimated 610,000 malaria-related deaths and 282 million malaria cases occurred globally in 2024 – an increase of about 9 million cases compared to 2023 [1]. The World Health Organization African Region, especially sub-Saharan Africa (SSA), continues to bear the greatest burden, with 11 countries accounting for about two thirds of global cases and deaths [1]. Despite progress in scaling up insecticide-treated nets (ITNs), chemoprevention, diagnostic testing, antimalarial treatment, and malaria vaccines, the burden of childhood malaria remains unevenly distributed across countries and population subgroups, which reflects both ecological transmission conditions and social and household factors [1–3].

Notably, ITNs can reduce malaria parasite prevalence and episodes of uncomplicated and severe malaria [4]. However, their population-level benefit depends on their general availability, allocation within households, and consistent use. The timely recognition of fever, care seeking, diagnostic testing, and treatment also depend on resources and health services available to households, as well as on caregivers’ knowledge and authority to act. More specifically, previous evidence from SSA has shown that maternal and household characteristics such as socioeconomic status, access to information, and participation in household or healthcare decisions are associated with care seeking or prompt treatment for childhood fever, which is a key symptom and treatment trigger for suspected malaria [5,6]. This would indicate that women’s position within households is relevant to the study of childhood malaria.

Women’s empowerment is a multidimensional process commonly conceptualised through access to resources, the capacity to exercise agency, and the achievement of valued outcomes [7]. Some multi-country studies have reported it to be associated with mortality among neonates, infants, and children under five years of age [8], and with child development, growth, nutrition, and nurturing-care practices [9]. Another quantitative study also found that empowerment measures were associated with maternal and child healthcare access and health outcomes, albeit with variations across measures and outcomes [10]. This evidence supports examining empowerment as a multidimensional correlate of child health, rather than as a single attribute.

Previous studies have also linked women’s empowerment to malaria-related health services, including care seeking for childhood fever, treatment use, and quality of malaria care [5,6]. However, evidence directly examining such multidimensional empowerment in relation to biomarker-confirmed malaria among children under five years of age remains limited. It is also unclear whether these associations are consistent across countries and household malaria-prevention practices.

In this study, we used nationally representative Demographic and Health Survey (DHS) data from 19 SSA countries surveyed between 2013 and 2022 to examine the associations of women’s empowerment, as measured by the domains of the Survey-based Women’s Empowerment (SWPER) Global index [11,12], with childhood malaria among children under five years of age. We also assessed variations in these associations by country and conducted exploratory analyses to determine whether they differed according to the children’s bed net utilisation.

## METHODS

We obtained data from the DHS, which conducted nationally representative household surveys that collect comparable population, health, and nutrition data using standardised questionnaires and biomarker protocols [13]. Most surveys used a two-stage stratified cluster sampling design with the first administrative units as urban and rural strata in each country’s region. In each stratum, a sample of clusters is selected independently with probability proportional to the contribution of that cluster’s population to the total population. After a complete household listing is conducted in each of the selected clusters, a fixed or variable number of households is selected by equal probability systematic sampling in the selected clusters. As this is a secondary data analysis, we confirm it aligns with the Journal of Global Health’s GRABDROP guidelines (Table S1 of the **Online Supplementary Document**) [14].

Ewerling and colleagues developed and validated the 14-item SWPER index using individual-level data from the DHS from sub-Saharan Africa [11] and subsequently expanded it as the SWPER Global framework for low- and middle-income countries, providing a standardised and comparable measure of women’s empowerment across survey settings [12].

We selected 19 of the 40 SSA countries included in the DHS which had complete women’s empowerment indicator data gathered through the SWPER Global and malaria biomarker test results for children under five years of age. We accessed the most recent DHS data of these countries, and for each survey, we linked the household members record, individual record, and children’s record files using standard DHS household, women, and child identifiers. The linked files provided malaria biomarker results, maternal SWPER items, child characteristics, and household characteristics. After registering with the DHS Program and receiving approval for the study’s data use request, we downloaded the de-identified datasets from the DHS Program website.

Children were eligible if they were aged 6–59 months, had valid results for both the malaria rapid diagnostic test and microscopy, could be linked to a surveyed mother for whom all three SWPER Global domain scores could be calculated, and had complete data for the prespecified covariates included in the primary model.

### Outcome variable

The outcome variable of interest was the malaria infection among children under five years of age, which was determined in the DHS based on positive rapid diagnostic tests or microscopy. Malaria biomarkers were collected by trained survey personnel following survey-specific standard protocols for rapid diagnostic testing and microscopy; details of the testing procedures and quality-control measures are provided in the DHS biomarker manuals and country survey reports [13,15]. Using the survey-specific biomarker variables, we classified children with a valid positive result as malaria-positive and those with a valid negative result as malaria-negative. Children with missing, refused, indeterminate, or otherwise unavailable results were excluded [16,17].

### Explanatory variable

Our key explanatory variable was women’s empowerment. Based on the published methods, we constructed the SWPER Global index from individual-level DHS items, thereby achieving the quantitative measurement of women’s empowerment. This indicator covers three domains [11,12]: attitude to violence, which comprises questions related to the women’s opinion on whether beating one’s wife is justified in some situations; social independence, which includes preconditions for empowerment, such as women’s access to information, educational attainment, age at first marriage and first child, and difference in age and education between women and their husbands; and decision-making, which is captured through three questions on who makes decisions in the household regarding the respondent’s healthcare, major expenses and visits to family and relatives. Empowerment levels were standardised according to the SSA region (eastern and southern Africa and western and central Africa) and we categorised the three domain scores and classified them as low, medium, and high empowerment using the published SWPER Global region-specific cut points [12].

### Child’s bed net utilisation in exploratory analyses

Child’s bed net utilisation was defined as the type of bed net a child slept under during the previous night, has been used as a standardised indicator within the DHS. For children under five years of age, this measure reflects access to and household allocation of bed nets [18]. Here, we classified utilisation as having no bed net, an untreated bed net, or an ITN, with children who slept under both an ITN and an untreated bed net included in the ITN category. Guided by the DHS indicator definitions and standard recode specifications, we harmonised across surveys using the available child-level bed net use and household net-roster information; consequently, no missing values remained in the final analytic sample.

### Covariates

Based on previous research on the associations between women’s empowerment and children’s health [5,6,19], we considered maternal age, maternal employment, child age, children’s sex, children’s birth order and interval, place of residence, and the household wealth index as our study covariates. The DHS household wealth index was a survey-specific composite measure of cumulative household living standards derived using principal component analysis of household assets, housing characteristics and materials, drinking-water sources, sanitation facilities, and other household amenities [19–21]. We recategorised the original five wealth quintiles as low (poorest and poorer), middle, and high (richer and richest).

### Data analysis

Within each country, we linked the household members record, individual record, and children’s record files obtained from the same DHS survey to combine malaria biomarker results, maternal SWPER items, child characteristics, and household characteristics, retaining only children with complete data on our variables and covariates of interest. We de-normalised the DHS household weights for the pooled analysis following DHS guidance and previous multi-country DHS studies [13,22]. For child *i* in country *c*, we calculated the weight as *ω_ic_* = (*HV*005*_ic_*/1,000,000) × (*N_c_*/*n_c_*), where *HV*005 was the DHS household sampling weight, which accounted for unequal household selection probabilities and household non-response. The variable was stored with six implied decimal places and was therefore divided by 1,000,000 before analysis. *N_c_* was the estimated national population of children under five years of age in the survey year and *n_c_* was the corresponding country-specific analytical sample. Country population estimates and sources are provided in Table S2 of the **Online Supplementary Document**. We then rescaled the resulting weights to an overall mean of one for likelihood-based modelling. Unweighted and weighted frequencies and percentages of all variables were calculated, and country-specific weighted malaria prevalence was mapped.

We examined associations between the three SWPER domains and childhood malaria positivity using binomial models with a logit link. First, we compared a weighted single-level logistic model with a weighted generalised linear mixed-effects model (GLMM) containing a random intercept for the primary sampling unit (PSU). Both models used the same sample, fixed-effect specification, and weights. We compared their fit using the Akaike and Bayesian information criteria, and selected the better-fitting weighted GLMM as the main model [22,23]. We fitted two specifications of the weighted GLMM: an exposure-only model containing the three SWPER domains simultaneously, and a fully adjusted model additionally containing the prespecified child-, maternal- and household-level covariates. We did not enter the survey year because each country contributed one survey, making the year collinear with country. We presented the results of the modelling as odds ratios (ORs) or adjusted odds ratios (aORs) with 95% confidence intervals (CIs). The model fit indicators included the PSU-level variance, intraclass correlation coefficient, Wald chi-squared statistic, log-likelihood, Akaike and Bayesian information criteria, and the number of PSUs; we otherwise assessed multicollinearity using variance inflation factors, with values below five considered acceptable.

As a sensitivity analysis, we replaced the categorical SWPER domains by their standardised continuous scores, with associations expressed per one-standard-deviation increase. All other model terms and weights in this analysis remained unchanged.

We evaluated country-specific associations using a two-stage approach [24]. First, we fitted fully adjusted weighted GLMMs with a PSU random intercept separately by country. Countries with fewer than 50 malaria-positive outcomes were reported as not estimable, with positive-outcome counts tabulated by country, domain, and empowerment level to identify sparse strata. Second, we pooled country-specific log-ORs for six prespecified comparisons: medium *vs*. low and high *vs*. low empowerment in each of the three SWPER domain, using random-effects meta-analysis with restricted maximum likelihood estimation and Hartung–Knapp inference. We reported the results of these analyses as random-effects pooled ORs, Cochran’s *Q*, *I*^2^, and *tau*^2^, and applied Benjamini–Hochberg (BH) adjustment separately to the heterogeneity tests and to all estimable country-specific contrasts. We displayed all country estimates, including imprecise and non-estimable results, together with pooled estimates within forest plots.

To examine the role of children’s bed net utilisation, we first added this variable as a covariate to the primary GLMM, we then fitted three domain-specific interaction models, each containing an interaction between bed net utilisation and one SWPER domain, while retaining the other two domains, covariates, country fixed effects, PSU random intercept, and weights. We assessed effect modification using one joint Wald test for the interaction terms in each domain-specific model and applied the Benjamini-Hochberg correction across the three tests. We presented the results as aORs and 95% CIs obtained as linear combinations from the pooled interaction models.

All tests were two-sided; a *P*-value <0.05 was used for prespecified primary analyses, and a BH-adjusted *P*-value <0.05 for exploratory country-specific and interaction analyses. We performed all analyses in *R*, version 4.3.2 (R Core Team, Vienna, Austria) within RStudio, version 2026.05.0 + 218 (Posit, PBC, Boston, Massachusetts, USA).

## RESULTS

Following screening, the final analytical sample included 79,739 children under five years of age in our analytical sample (Figure S1 of the **Online Supplementary Document**). The mean age was 31.67 months (standard deviation = 15.61); almost half lived in low-wealth index households (n = 37,103, 46.53%) and slept under ITN the night before the survey (n = 42,185, 52.90%). In addition, low empowerment was most prevalent in the decision-making domain (50.66%) and least prevalent in the domain of attitude to violence (33.59%) (**Table 1**).

**Table 1:** Characteristics of the children (n = 79,739) in 19 sub-Saharan African countries, n (%)

	Total sample	Weighted sample*
**Maternal age in years**		
15–24	18,289 (22.94)	18,523 (23.23)
25–34	39,656 (49.73)	40,090 (50.28)
35–49	21,794 (27.33)	21,126 (26.49)
**Maternal employment**		
No	25,846 (32.41)	24,406 (30.61)
Yes	53,893 (67.59)	55,333 (69.39)
**Residence**		
Urban	26,675 (33.45)	27,769 (34.82)
Rural	53,064 (66.55)	51,970 (65.18)
**Children’s age in months**	31.67 (15.61)	31.57 (15.57)
**Children’s sex**		
Male	40,339 (50.59)	40,239 (50.46)
Female	39,400 (49.41)	39,500 (49.54)
**Birth order**		
1	14,210 (17.82)	14,169 (17.77)
2–4	39,465 (49.49)	39,335 (49.33)
≥5	26,064 (32.69)	26,234 (32.90)
**Birth interval**		
≤24 months	15,543 (19.49)	16,844 (21.12)
>24 months	64,196 (80.51)	62,895 (78.88)
**Household wealth index**		
Low	37,103 (46.53)	34,258 (42.96)
Medium	16,523 (20.72)	16,131 (20.23)
High	26,113 (32.75)	29,350 (36.81)
**Children’s bed net utilisation**		
No bed net	35,163 (44.10)	34,129 (42.80)
Untreated bed net	2,391 (3.00)	1,868 (2.34)
ITN	42,185 (52.90)	43,742 (54.86)
**Attitude to violence**		
Low	26,781 (33.59)	26,902 (33.74)
Medium	8,035 (10.07)	7,233 (9.07)
High	44,923 (56.34)	45,604 (57.19)
**Social independence**		
Low	37,830 (47.44)	37,322 (46.80)
Medium	19,523 (24.48)	18,574 (23.29)
High	22,386(28.07)	23,843 (29.90)
**Decision-making**		
Low	40,394 (50.66)	41,512 (52.06)
Medium	34,592 (43.38)	33,826 (42.42)
High	4,753 (5.96)	4,401 (5.52)

*Weighted counts were calculated using de-normalised sampling weights rescaled to an overall mean of 1.

### Distribution of malaria prevalence among children in 19 SSA countries

After applying the survey weights, the pooled malaria prevalence was 27.95%. Weighted childhood malaria prevalence varied substantially, ranging from 0.41% in The Gambia and 0.62% in Mauritania to 48.06% in Benin. The highest weighted prevalence was observed in Benin (48.06%), Togo (44.54%), Burundi (40.25%), and Côte d’Ivoire (40.03%), and the lowest in The Gambia (0.41%), Mauritania (0.62%), and Rwanda (2.86%) (**Figure 1**).

**Figure 1:**
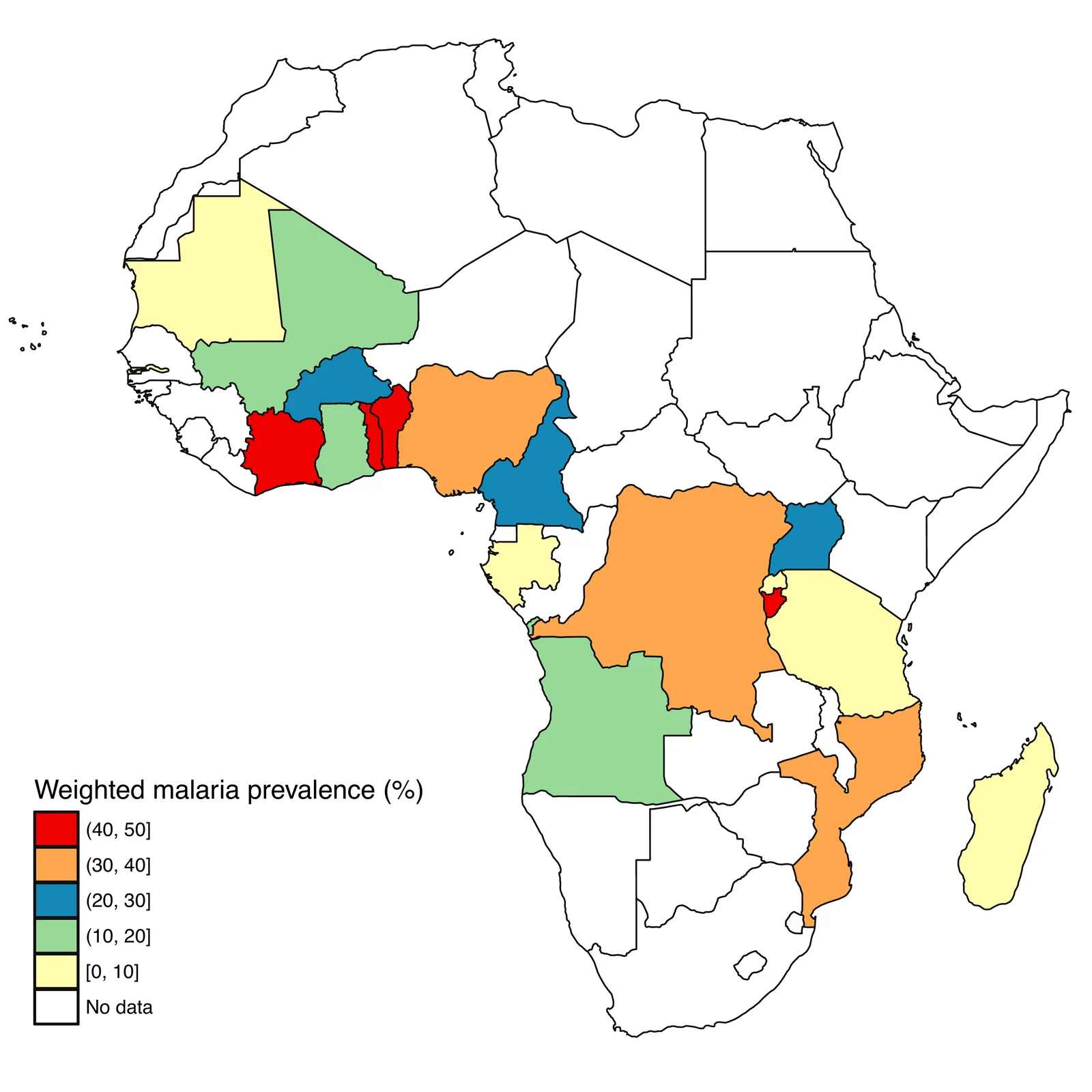
Weighted prevalence of childhood malaria in 19 sub-Saharan African countries (%). White areas indicate countries not included in the analysis.

### Association between women’s empowerment and childhood malaria

In the fully adjusted weighted GLMM, compared with low empowerment, both medium (aOR = 0.85; 95% CI = 0.78–0.92) and high (aOR = 0.85; 95%CI = 0.80–0.90) empowerment in the attitude to violence domain were associated with lower odds of malaria positivity. For social independence, high empowerment was associated with lower odds of malaria positivity (aOR = 0.79; 95% CI = 0.74–0.84), while we found no evidence of an association for medium social independence (aOR = 0.96; 95% CI = 0.91–1.02). Similarly, high decision-making empowerment was associated with lower odds of malaria positivity (aOR = 0.89; 95% CI = 0.80–0.99), while we found no evidence of an association for medium decision-making empowerment (aOR = 0.96; 95% CI = 0.91–1.01). We observed a strongest adjusted association for high social independence, corresponding to approximately 21% lower odds of malaria positivity (**Table 2**; Figure S2 of the **Online Supplementary Document**).

**Table 2:** Association between women’s empowerment and childhood malaria in sub-Saharan African countries using weighted generalised linear mixed-effects model

	cOR (95% CI)	*P*-value	aOR (95% CI)*	*P*-value
**Fixed effect estimates**				
Attitude to violence				
*Low*	ref		ref	
*Medium*	0.82 (0.75–0.89)		0.85 (0.78–0.92)	
*High*	0.79 (0.75–0.83)		0.85 (0.80–0.90)	
Social independence				
*Low*	ref		ref	
*Medium*	0.85 (0.80–0.89)		0.96 (0.91–1.02)	
*High*	0.59 (0.56–0.63)		0.79 (0.74–0.84)	
Decision-making				
Low	ref		ref	
Medium	0.92 (0.88–0.97)		0.96 (0.91–1.01)	
High	0.87 (0.79–0.97)		0.89 (0.80–0.99)	
**Covariates**				
Maternal age				
*15–24*			ref	
*25–34*			0.98 (0.92–1.05)	
*35–49*			0.91 (0.84–1.00)	
Maternal employment				
*No*			ref	
*Yes*			0.96 (0.91–1.02)	
Residence				
*Urban*			ref	
*Rural*			3.01 (2.72–3.33)	
Children’s age in months			1.02 (1.02–1.03)	
Children’s sex				
*Male*				
*Female*			0.99 (0.95–1.03)	
Birth order				
*1*			ref	
*2–4*			1.08 (1.01–1.16)	
*≥5*			1.25 (1.14–1.37)	
Birth interval				
*≤24 months*			ref	
*>24 months*			0.97 (0.92–1.02)	
Household wealth index				
*Low*			ref	
*Medium*			0.68 (0.64–0.72)	
*High*			0.37 (0.34–0.40)	
Country				
*Angola*			ref	
*Benin*			10.29 (8.01–13.22)	
*Burkina Faso*			2.32 (1.81–2.99)	
*Burundi*			4.27 (3.31–5.51)	
*Cameroon*			2.06 (1.58–2.67)	
*Côte d’Ivoire*			6.01 (4.73–7.65)	
*DRC*			4.19 (3.34–5.27)	
*Gabon*			0.97 (0.57–1.66)	
*Gambia*			0.02 (0.01–0.12)	
*Ghana*			1.15 (0.89–1.49)	
*Madagascar*			0.21 (0.15–0.27)	
*Mali*			0.74 (0.56–0.98)	
*Mauritania*			0.02 (0.01–0.06)	
*Mozambique*			1.86 (1.45–2.38)	
*Nigeria*			5.31 (4.34–6.48)	
*Rwanda*			0.10 (0.07–0.15)	
*Tanzania*			0.16 (0.12–0.21)	
*Togo*			6.81 (5.07–9.15)	
*Uganda*			2.06 (1.63–2.60)	
**Random effect parameters and model statistics**				
PSU random-intercept variance			2.158	
ICC			0.396	
Wald chi-squared statistic			5,350.33	
**Model fit**				
Log-likelihood			−34,229.97	
AIC			68,533.95	
BIC			68,877.55	
Number of PSU clusters			11,110	

AIC – Akaike information criterion, aOR – adjusted odds ratio, BIC – Bayesian information criterion, CI – confidence interval, cOR – crude odds ratio, ICC – intraclass correlation coefficient, PSU – primary sampling unit, ref – reference

*The adjusted model included all three SWPER domains, maternal age, maternal employment, child age, child sex, birth order, birth interval, residence, household wealth index and country fixed effects, with a PSU random intercept.

Sensitivity analysis treating the three SWPER domain scores as continuous variables yielded findings consistent with those of the primary analysis. In the adjusted weighted GLMM, each one standard deviation increase in the empowerments regarding attitudes to violence (aOR = 0.925; 95% CI = 0.903–0.948, *P* < 0.001), social independence (aOR = 0.844; 95% CI = 0.819–0.869, *P* < 0.001), and decision-making (aOR = 0.949; 95% CI = 0.925–0.973, *P* < 0.001) was associated with lower odds of malaria positivity (Table S4 of the **Online Supplementary Document**).

### Country-specific association between women’s empowerment and childhood malaria across 19 different SSA countries

Country-specific analyses showed substantial variation in outcome counts, ranging from 10 malaria-positive children in The Gambia and 22 in Mauritania to 3,796 in Nigeria (Table S3 of the **Online Supplementary Document**). All six contrasts for The Gambia and Mauritania were reported as not estimable, meaning that these countries had fewer than 50 malaria-positive outcomes, precluding stable fully adjusted multivariable estimation.

Random-effects pooling showed inverse associations only for medium (aOR = 0.86; 95% CI = 0.77–0.97) and high (aOR = 0.85; 95%CI = 0.78–0.92) attitude to violence empowerment; the 95% CIs for all four social independence and decision-making contrasts included 1.

After BH correction across the six heterogeneity tests, we observed significant between-country heterogeneity for medium social independence (*I*^2^ = 49.3%, *Q* = 33.00, *P* = 0.007, Benjamini–Hochberg-adjusted *P* (*P*_BH_) = 0.014) and high social independence (*I*^2^ = 62.3%, *Q* = 44.81, *P* < 0.001, *P*_BH_ < 0.001). We also detected heterogeneity for medium (*I*^2^ = 58.5%, *Q* = 37.05, *P* = 0.002, *P*_BH_ = 0.006) and high decision-making empowerment (*I*^2^ = 49.9%, *Q* = 32.23, *P* = 0.009, *P*_BH_ = 0.014). Neither the medium *vs*. low attitude to violence contrast (*Q = *24.00, *I*^2^* = *24.6%,* P = 0.089, P*_BH_* = *0.089) nor the high *vs*. low contrast (*Q = *24.09, *I*^2^* = *28.3%, *P = *0.087, *P*_BH_* = *0.089) showed evidence of between-country heterogeneity after correction.

Of 114 planned country-specific contrasts, 12 were not estimable, leaving 102 for analysis across 17 countries. Twenty-one contrasts had nominal *P*-values <0.05, of which six remained significant after BH correction. These comprised medium attitude-to-violence empowerment in Mozambique (aOR = 0.47; 95% CI = 0.28–0.77, *P*_BH_ = 0.044) and high attitude-to-violence empowerment in Nigeria (aOR = 0.80; 95% CI = 0.70–0.92, *P*_BH_ = 0.044) and Uganda (aOR = 0.58; 95% CI = 0.46–0.74, *P*_BH_ < 0.001). Medium (aOR = 0.76; 95% CI = 0.65–0.89, *P*_BH_ = 0.022) and high (aOR = 0.59; 95% CI = 0.50–0.69, *P*_BH_ < 0.001) social independence remained significant in Nigeria, while medium decision-making empowerment remained significant in Burundi (aOR = 0.77; 95% CI = 0.65–0.91, *P*_BH_ = 0.044) (**Figure 2**).

**Figure 2:**
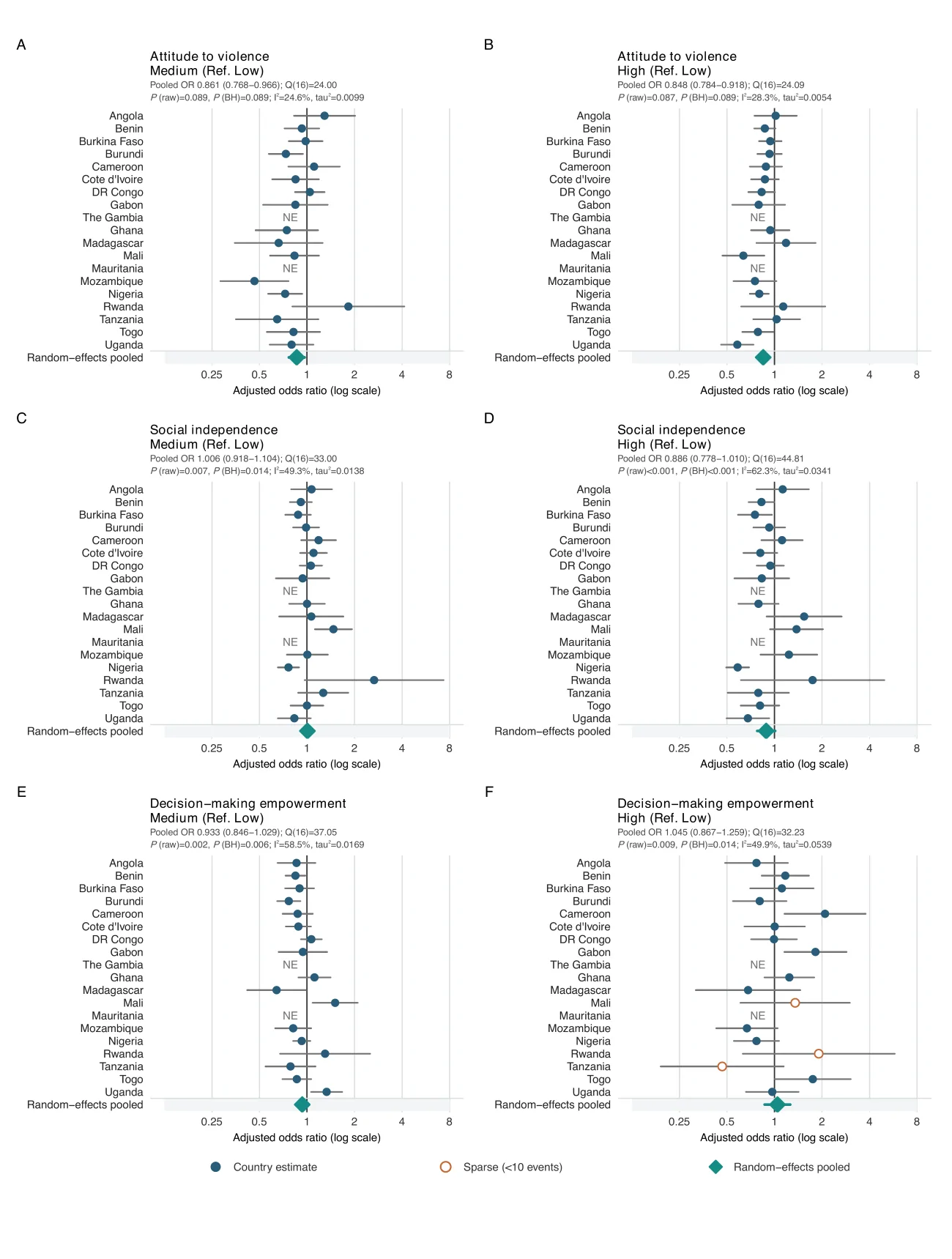
Country-specific associations between women’s empowerment and childhood malaria. **Panel A.** Medium *vs*. low attitude-to-violence empowerment. **Panel B.** High *vs*. low attitude-to-violence empowerment. **Panel C.** Medium *vs* low social independence. **Panel D.** High *vs*. low social independence. **Panel E.** Medium *vs*. low decision-making empowerment. **Panel F.** High *vs*. low decision-making. BH – Benjamini–Hochberg, CI – confidence interval, NE – not estimable, OR – odds ratio.

### Exploratory adjusted and interaction analyses of children’s bed net utilisation

Additional adjustment for the children’s bed net utilisation produced little change in the associations between women’s empowerment and childhood malaria. For medium and high empowerment, respectively, the aORs were 0.85 (95% CI = 0.79–0.92) and 0.85 (95% CI = 0.80–0.90) for attitude to violence, 0.96 (95% CI = 0.91–1.02) and 0.79 (95% CI = 0.74–0.85) for social independence, and 0.96 (95% CI = 0.91–1.01) and 0.88 (95% CI = 0.79–0.99) for decision-making. Interaction analyses revealed that the associations between attitude to violence and childhood malaria varied by children’s bed net utilisation (BH-adjusted interaction *q* = 0.017). Medium and high empowerment were associated with lower malaria odds among children without a bed net, with respective aORs of 0.79 (95% CI = 0.70–0.90) and 0.86 (95% CI = 0.80–0.93) and among ITN users, with aORs of 0.86 (95% CI = 0.77–0.96) and 0.83 (95% CI = 0.77–0.89). Conversely, medium empowerment was associated with higher odds in the untreated bed net group (aOR = 1.95; 95% CI = 1.13–3.36). Decision-making also showed heterogeneity (*P*_BH_ = 0.010), where medium *vs*. low empowerment was inversely associated with malaria among children without a bed net (aOR = 0.91; 95% CI = 0.85–0.98) and untreated bed net users (aOR = 0.53; 95% CI = 0.37–0.77), but not among ITN users (aOR = 1.02; 95% CI = 0.95–1.08). There was no evidence of interaction for social independence (*P*_BH_ = 0.625) (**Figure 3**; Table S6 of the **Online Supplementary Document**).

**Figure 3:**
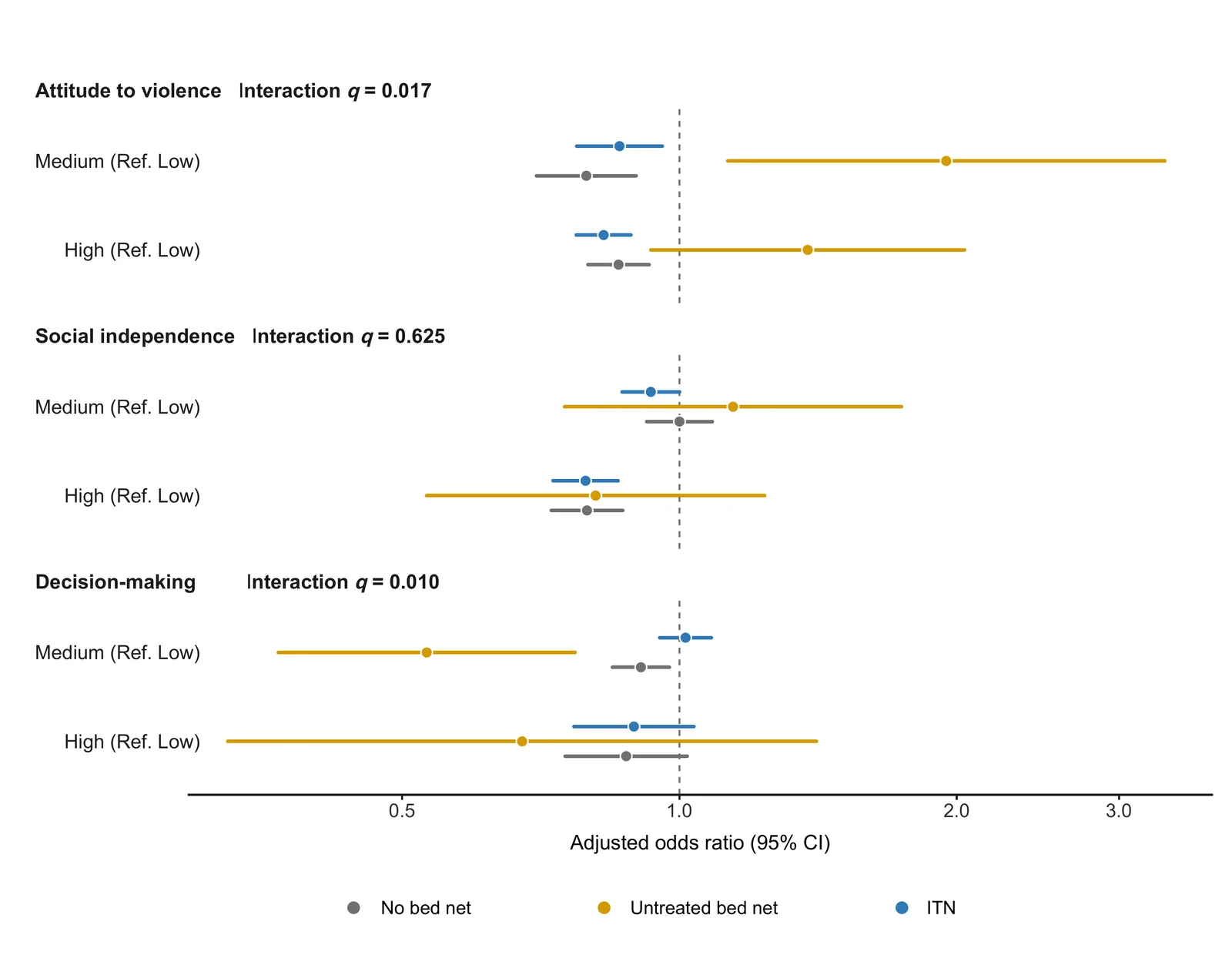
Interaction analysis between women’s empowerment and childhood malaria according to the children’s bed net utilisation. CI – confidence interval, ITN – insecticide-treated net.

## DISCUSSION

In this pooled analysis of nationally representative DHS data from 19 SSA countries, we found that selected dimensions of women’s empowerment were associated with childhood malaria test positivity among children under five years of age. We also observed inverse associations for medium and high empowerment in attitude to violence, high social independence, and high decision-making, and no associations for medium social independence or medium decision-making. Sensitivity analyses using continuous SWPER Global scores broadly supported this domain-specific result, while country-specific analyses showed that some associations varied across national settings.

To our knowledge, this is the first multi-country study to examine the association between multidimensional women’s empowerment, measured by the SWPER Global index, and biomarker-confirmed malaria among children, while also assessing between-country heterogeneity and variation according to the children’s bed net utilisation. Previous studies have linked women’s empowerment with maternal and child healthcare access and a range of child health outcomes, although the strength and direction of these associations have varied across empowerment dimensions and settings [6–8]. In a malaria-related study, Lewis and colleagues found that multidimensional empowerment was associated with care seeking for childhood fever and with aspects of care quality among children with malaria [5]. Our findings complement this evidence by identifying associations between multidimensional women’s empowerment and malaria test positivity – a child health outcome distinct from care seeking and treatment.

The inverse associations observed for the attitude-to-violence domain support the hypothesis that gender-related norms within households may be relevant to childhood malaria. This SWPER domain reflects rejection of circumstances used to justify wife beating and is commonly regarded as an indicator of gender norms and women’s intrinsic agency [11,12]. Previous research has linked rejection of intimate partner violence with greater use of maternal and child health interventions and improved child survival [8,11]. One possible explanation is that rejection of gender-inequitable norms may accompany greater communication between partners and participation in child-health decisions, pathways proposed in previous work on empowerment and health [9,25].

High social independence showed the clearest inverse association in both categorical and continuous analyses. This finding supports the conceptual proposition that the social and informational resources represented by this domain may be relevant to child health. The social independence domain of the SWPER index combines educational attainment, access to information, age at marriage and first birth, and age and educational differences between partners. Previous multi-country studies have linked women’s education, information access, and empowerment-related resources with child-health practices and outcomes [5,6,9,10]. Multi-country DHS analyses have linked women’s empowerment and social resources with child development, nutrition and nurturing care [9]. Socioeconomic circumstances are also strongly patterned with childhood malaria risk in SSA [2,3]. The association we observed for high, but not medium social independence suggests that high scores may identify a broader combination of favourable household and community circumstances.

The decision-making domain showed a more modest pattern, with an inverse association at the high level, but no association at the medium level. This pattern is consistent with previous evidence that women’s participation in decisions may facilitate the use of resources for child health. Decisions concerning healthcare, major household purchases, and visits to relatives may influence the timing of care seeking, access to preventive products, and the allocation of household resources. One study has similarly found associations between maternal decision-making power and care-seeking for acutely ill children [26], while another identified links of empowerment with care utilisation and quality [5]. However, participation in a decision may differ from control over resources or the capacity to implement a preferred action. The less consistent pattern for this domain may, therefore, reflect differences in the form and practical meaning of household decision-making across families and countries.

In our country-specific analyses, we identified heterogeneity in both social independence contrasts and both decision-making contrasts, while the associations for attitude to violence were comparatively consistent across countries. The clearest country-specific findings included inverse associations for medium and high empowerment of social independence in Nigeria, high empowerment of attitude to violence in Nigeria and Uganda, medium empowerment of attitude to violence in Mozambique, and medium empowerment of decision-making in Burundi. Nigeria, Uganda, and Mozambique were among the five countries accounting for more than half of malaria cases in the World Health Organization African Region in 2024, indicating a high malaria burden in all three countries, although transmission intensity and spatial distribution differ within and between countries [1]. In Nigeria, community gender norms have been shown to modify the health relevance of women’s status, supporting the possibility that social independence and attitude to violence acquire different practical meanings across communities [27]. In Mozambique, childhood malaria prevalence varies markedly across provinces and is patterned by rural residence, household wealth, and maternal education, which shows how local malaria ecology and socioeconomic conditions may intersect [28]. Our findings regarding Burundi are also consistent with a previous study linking maternal decision-making power to care-seeking for acutely ill children in the country [26]. Collectively, these findings suggest that the implications of a given SWPER domain may depend on the combination of gender norms, socioeconomic opportunities, malaria transmission, and health service environments within each country [10–12].

The exploratory analyses provided additional insight into the household prevention context of the observed associations. The associations for attitude to violence and decision-making differed across bed net utilisation, while the association for social independence did not. Decision-making is a relatively proximal expression of agency over healthcare and household resources and may influence how available nets are obtained or allocated. For example, a study from Uganda indicated that when household nets are insufficient, their actual allocation may differ from stated preferences to protect young children [18]. While attitude to violence reflects women’s acceptance or rejection of gender-inequitable norms and may be related to their capacity to negotiate the use of household preventive resources [11,12]. Its association with childhood malaria may, therefore, vary according to the children’s bed net utilisation. The reversed estimate for medium attitude-to-violence empowerment among untreated-net users may also reflect selective net allocation and unmeasured differences in net availability, condition, comfort, or perceived malaria risk, all of which can influence reported net use [29].

In contrast, social independence incorporates education, access to information, and life-course characteristics. These broader resources may relate to malaria positivity by way of health knowledge, socioeconomic circumstances, and access to services, rather than depending strongly on the children’s bed net utilisation on a single night. Education, media exposure, and household resources have themselves been associated with ITN utilisation in East Africa [30]. The absence of interaction for social independence is, therefore, compatible with a comparatively similar association across the observed bed net utilisation categories, although these explanations remain hypotheses. Longitudinal studies incorporating repeated measures of net access, type, condition and use, together with seasonal and entomological data, are needed to confirm these interactions and distinguish household allocation processes from differences in malaria exposure.

This study has several strengths. We combined harmonised, nationally representative data from 19 countries, used biomarker-based malaria outcomes, and measured women’s empowerment using the validated, multidimensional SWPER Global index. We accounted for sampling weighting, clustering within PSUs, and country fixed effects. Analyses using SWPER scores assessed sensitivity to categorical cut points, while multiplicity-adjusted country-specific and interaction analyses examined contextual variation.

Several limitations, however, should be considered. First, the cross-sectional design and concurrent measurement of women’s empowerment, bed net utilisation, and malaria status limit temporal and causal inference. Second, residual confounding may have affected our results because altitude, local transmission intensity, rainfall, season, interview month, intervention coverage, and health system characteristics were not consistently available in the pooled dataset. Third, the DHS wealth index measures relative socioeconomic position within each country and survey, rather than an absolute level of wealth that is directly comparable across settings [21]. Fourth, the SWPER Global index is primarily available for partnered women, which limits generalisability to children of non-partnered mothers and other caregivers. Maternal education was not entered in the model as a separate covariate because it is an explicit component of the SWPER social independence domain; consequently, the contribution of education cannot be separated from the composite domain in this analysis. Furthermore, empowerment indicators, employment, and bed net utilisation were self-reported and may have been affected by recall, social desirability, and cultural interpretation; bed net utilisation referred to the night preceding the interview and may not represent longer-term use. Finally, estimates from the relatively small untreated-bed-net group were less precise, and country-specific models could not be estimated reliably for countries with very few malaria-positive outcomes. Because the country-specific and interaction analyses involved multiple comparisons and some sparse strata, these findings should be considered exploratory and require independent confirmation.

## CONCLUSIONS

Our analysis provides evidence that selected dimensions of women’s empowerment could be associated with lower odds of biomarker-confirmed malaria among children under five years of age across 19 SSA countries. We observed the clearest association for high empowerment of social independence, with inverse associations for medium and high empowerment of attitude to violence and for high empowerment of decision-making. Country-specific analyses identified inverse associations for selected contrast in Nigeria, Uganda, Mozambique, and Burundi, while attitude-to-violence associations were comparatively consistent across settings. Exploratory analyses showed that the associations for attitude to violence and decision-making differed according to the children’s bed net utilisation, with no such variation for social independence. These findings support considerations of gender-related social conditions and household prevention practices alongside established malaria-control interventions. Longitudinal and mixed-methods studies incorporating repeated measures of empowerment and bed-net use, together with subnational transmission and intervention data, are needed to clarify the temporal relationships and context-specific mechanisms underlying these associations.

## Additional material


Online Supplementary Document

## Data Availability

**Data availability:** The data used in this study are publicly available from the DHS Program team *via* the following link: https://dhsprogram.com/.
