# Supplementary material for: Women’s empowerment and childhood malaria in sub-Saharan Africa: evidence from DHS, 2013–2022
Source: J Glob Health. 2026 Oct 23;16:04369. doi: 10.7189/jogh.16.04369 (PMC13693444; doi:10.7189/jogh.16.04369)
Supplement: Supplementary file 1 [file jogh-16-04369-s001.pdf]

Supplement to: Pei Z, Ru X, Yu X, Zhou S, Ong SK, El Rabbat M, Xu M. Women's empowerment and childhood malaria in sub-Saharan Africa: evidence from DHS, 2013–2022. J Glob Health. 2026;16:04369.

Table S1. Adherence to JoGH's GRABDROP guidelines items……………………..2

Women's empowerment definition…………………………………………………5

Table S2. Country-specific target populations used to de-normalize the DHS household sampling weights……………………………………………………………………6

Figure S1. The process of sample selection…………………………………………...7

Table S3. The distribution of malaria positive cases in 19 sub-Saharan African countries ………………….…………………………………..………………………8

Figure S2. Forest plot of the association between women's empowerment and the childhood malaria positivity in 19 sub-Saharan African countries..……………………9

Table S4. Sensitivity analysis of the main model………..……………………………9

Table S5. Associations between women's empowerment and childhood malaria before and after additional adjustment for the child's bed net utilization……….....................10

Table S6. Stratum-specific associations between women's empowerment and childhood malaria according to the child's bed net utilization…….......................11

Table S1. Adherence to JoGH's GRABDROP guidelines items.

**JoGH guideline items**
1. Please list all papers published by each co-author in previous 3 years that were based on secondary analysis of a big data repository
[1] **Zhou S**, Liang Z, Li Q, Song S, Wang Z, **Xu M**, Jin Y, Zheng ZJ. Association of cumulative average sensory impairments with cognitive function and depressive symptoms: Two prospective cohort studies. J Affect Disord. 2025 Jan 15;369:16-24.<br>[2] Xie S, Zhang L, Zhang Y, Zheng ZJ, Li J, **Zhou S**. Global, regional, and national burden of ischemic heart disease and ischemic stroke and their risk factors in youths and young adults aged 15-39 years (1990-2021): a comparative analysis of risk factors from global burden of disease study 2021. Glob Health Action. 2025;18(1):2560711.<br>[3] Xie SW, Zhang L, Zheng ZJ, Li J, **Zhou S**. Associations of childhood smoking initiation age with cardiometabolic multi-morbidity and mortality: a multi-national cohort study. BMC Public Health. 2026;26(1):590. Published 2026 Jan 20.<br>[4] Hong C, Dai W, **Xu M**, Luo Y. Global trends and regional differences in the burden of HIV/AIDS attributed to intimate partner violence among females in 204 countries and territories, 1999-2019: An analysis of the global burden of disease study. Psychol Trauma. 2025 Nov;17(8):1641-1650.<br>[5] Global Cardiovascular Risk Consortium; Magnussen C, Alegre-Diaz J, Al-Nasser LA, Amouyel P, Aviles-Santa L, Bakker SJL, Ballantyne CM, Bernabé-Ortiz A, Bobak M, Boffetta P, Brenner H, Brunström M, Can G, Carrillo-Larco RM, Checkley W, Dallongeville J, De Bacquer D, de Gaetano G, de Lemos JA, di Carluccio E, Dobson A, Donfrancesco C, Dörr M, d'Orsi E, Drygas W, Dullaart RPF, Engström G, Ferrario MM, Ferrières J, Figtree GA, Gaye B, Ghayour-Mobarhan M, Goldbourt U, Gonzalez C, Gossling A, Grassi G, Gupta PC, He J, Hodge AM, Hozawa A, Hveem K, Iacoviello L, Ikram MK, Inoue M, Irazola V, Jobe M, Jousilahti P, Kaleebu P, Kavousi M, Kee F, Khalili D, Klotsche J, Koenig W, Kontsevaya A, Kowlessur S, Kuri-Morales P, Kuulasmaa K, Kweon SS, Lackner KJ, Landmesser U, Leistner DM, Leiva Sisnieguez CE, Leong D, Lind L, Linneberg A, Lorenz T, Lyngbakken MN, Malekzadeh R, Malyutina S, Mathiesen

EB, McElduff P, Melander O, Metspalu A, Miranda JJ, Moitry M, Mugisha J, Munzinger J, Nalini M, Nambi V, Nilsson PM, Ninomiya T, Omland T, **Ong SK**, Oppermann K, Pajak A, Palmieri L, Panagiotakos D, Park SK, Pednekar MS, Perianayagam A, Peters A, Poustchi H, Prabhakaran D, Prentice AM, Prescott E, Quyyumi A, Risérus U, Sakata S, Salazar M, Salomaa V, Sans S, Sattler ELP, Schöttker B, Schutte AE, Sepanlou SG, Sharma SK, Shaw J, Simons LA, Söderberg S, Tamosiunas A, Tapia-Conyer R, Thorand B, Tunstall-Pedoe H, Tuomilehto J, Twerenbold R, Vanuzzo D, Veronesi G, Wannamethee SG, Watanabe M, Weimann J, Wild PS, Yao Y, Zeng Y, Ziegler A, Ojeda FM, Blankenberg S; The Global Cardiovascular Risk Consortium. Global Effect of Cardiovascular Risk Factors on Lifetime Estimates. N Engl J Med. 2025 Jul 10;393(2):125-138.

[6] Girardi F, Nyangasi M, Callender C, Ng M, Narita S, Torode J, AlSawahli H, **Ong SK**, Gnangnon F, McCormack V, Shelpai W, Rizu, Mahmoud L, Wang H, Ilbawi AM. Global breast cancer survival estimates in 2017-2021 to advance the WHO Global Breast Cancer Initiative. Nat Med. 2026 Jul 8.

[7] Rehm J, Amul GGH, Assanangkornchai S, Chaiyasong S, Correia D, Hairi NN, Hanafi E, Hassan AS, Htet KKK, Low WY, Medina JRC, Nontarak J, **Ong SK**, Rovira P, Siste K, Sornpaisarn B, Sychareun V, Tong WT, Vichitkunakorn P, Yi S, Zayar NN, Shield K. Defining exposure for estimating the global burden of alcohol consumption: plausibility testing of WHO methodology using ASEAN countries as a case study. Subst Abuse Treat Prev Policy. 2026 Jan 30;21(1):18.

2. Please explain the key elements of your study design and the use of the available datasets that make your study an original scientific contribution

This study combines nationally representative DHS data from 19 sub-Saharan African countries to examine three standardized SWPER Global domains in relation to biomarker-confirmed malaria among children under 5 years. We conducted the pooled weighted GLMM with country fixed effects and a country-unique PSU random intercept; continuous-score sensitivity analyses; prespecified country-specific models followed by random-effects synthesis and formal heterogeneity testing; and exploratory evaluation of whether associations differ by the child’s bed net utilization.

3. Please list all publications that addressed similar research questions in the same dataset and indicate where you cited them in your paper
Lewis et al. (J Glob Health. 2022;12:04025), a 16-country DHS analysis of women’s empowerment, care seeking, and quality of malaria care, is cited in the Background and Discussion (current reference 3; main-manuscript lines 73, 100–102, and 403–405). Chilot et al. (PLoS One. 2023;18:e0285265), a pooled analysis of malaria prevalence and risk factors in 13 sub-Saharan African countries, is cited in the outcome definition (current reference 13; lines 148–154). Anjorin et al. (Malar J. 2023;22:55), an 11-country analysis of malaria profiles and socioeconomic predictors, is cited in the Discussion (current reference 23; lines 432–433). Eom et al. (SSM Popul Health. 2024;26:101651), a 28-country analysis of women’s empowerment and child anthropometric failures, is cited in the covariate rationale (current reference 14; lines 185–189). None addresses the same combination of SWPER Global domains, biomarker-confirmed childhood malaria, country heterogeneity, and bed net utilization interaction analyses.
4. Please explain how you addressed multiple testing through an appropriately rigorous statistical threshold and indicate this in the methods section
Benjamini–Hochberg adjustment was applied separately to the six country-level heterogeneity tests, all estimable country-specific exposure contrasts, and the three bed-net interaction tests. Raw and adjusted P values were reported, and BH-adjusted $P<0.05$ was used for the exploratory analyses (main-manuscript lines 236–256).
5. Please declare to what extent have AI chatbots been used in developing your paper and to which parts of the paper did they contribute
In this study, the AI was supervised by the author and was used to assist with the English language polishing and consistency check of the content of this manuscript. The acquisition of DHS data, data analysis, making independent scientific decisions, and the full text writing were all independently completed by the author, and the author assumes full responsibility for the entire manuscript.

Women's empowerment definition

Women's empowerment is defined as the ability of a woman to claim enabling resources, exercise voice and agency, and act on desires to transform her own life in contexts where this ability has been denied. In our study, women's empowerment was measured by SWPER Global. This index aims to access the individual level of women's empowerment and it includes the following 14 items in three domains:

Item
**Attitude to violence**
1. Beating not justified if wife goes out without telling husband
2. Beating not justified if wife neglects the children
3. Beating not justified if wife argues with husband
4. Beating not justified if wife refuses to have sex with husband
5. Beating not justified if wife burns the food
**Social independence**
6. Frequency of reading newspaper or magazine
7. Woman education in completed years of schooling
8. Age of woman at first birth
9. Age of woman at first cohabitation
10. Age difference: woman's minus husband's age
11. Education difference: woman's minus husband's years of schooling
**Decision-making**
12. Who usually decides on respondent's health care
13. Who usually decides on large household purchases
14. Who usually decides on visits to family or relatives

Table S2. Country-specific target populations used to de-normalize the DHS household sampling weights

Country	Reference year	Sample size, n_c	Estimated population aged under 5 years, N_c[*]	N_c/n_c
Angola	2015	4,295	4,571,500	1,064.4
Benin	2017	5,354	1,806,856	337.5
Burkina faso	2021	5,018	3,045,842	607.0
Burundi	2016	4,902	1,922,408	392.2
Cameroon	2018	3,299	3,608,622	1,093.9
Cote d'ivoire	2021	3,850	4,081,347	1,060.1
DRC	2013	6,457	12,517,578	1,938.6
Gabon	2020	3,140	288,052	91.7
Gambia	2020	3,096	337,415	109.0
Ghana	2022	3,480	3,776,503	1,085.2
Madagascar	2021	4,167	3,952,780	948.6
Mali	2021	3,735	3,577,725	957.9
Mauritania	2020	3,826	676,389	176.8
Mozambique	2018	2,901	4,519,134	1,557.8
Nigeria	2018	9,599	29,963,358	3,121.5
Rwanda	2020	2,864	1,642,187	573.4
Tanzania	2022	3,704	9,483,720	2,560.4
Togo	2014	2,645	1,072,892	405.6
Uganda	2016	3,407	6,125,344	1,797.9

[*]Footnote 1. Source from United Nations, Department of Economic and Social Affairs, Population Division. World Population Prospects 2024, Online Edition. New York: United Nations; 2024. Available: https://population.un.org/wpp/ (Accessed: 29 July 2026).

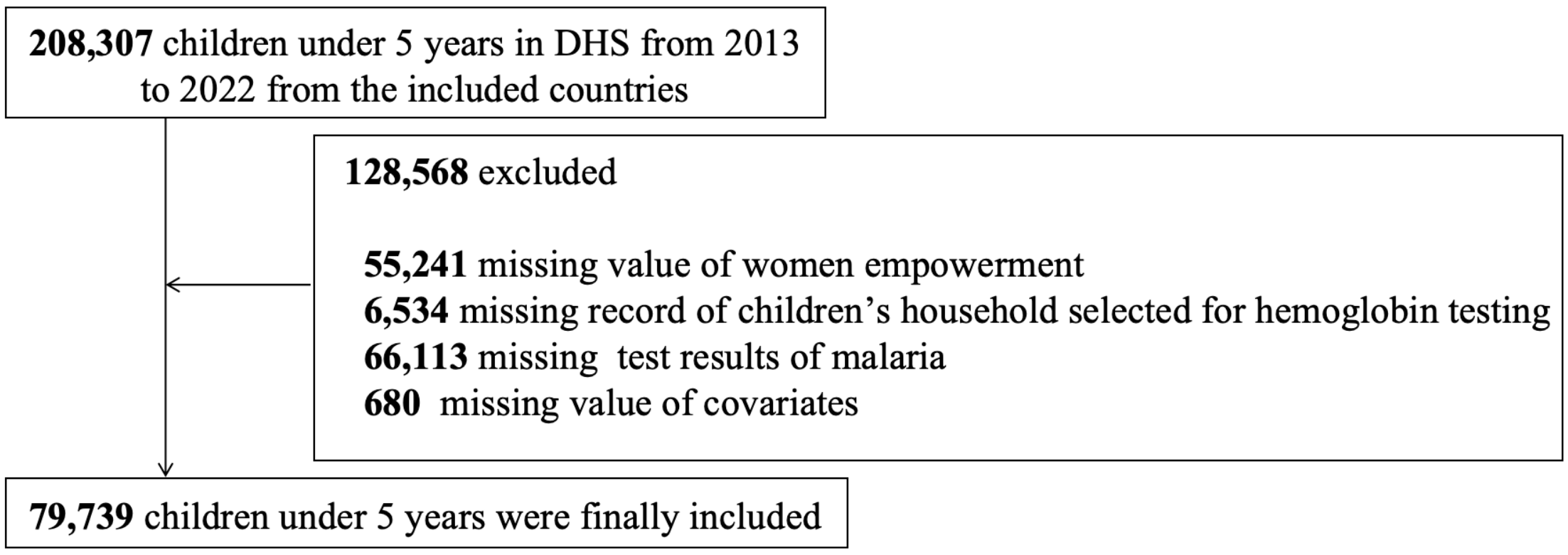

Figure S1. The process of sample selection.

Table S3. The sample size and distribution of malaria positive cases in 19 sub-Saharan African countries

Country	Year	Sample size, n	Malaria positive cases, n (%)	Weighted malaria-positive*, n (%)
Angola	2015	4,295	741 (17.25%)	551 (14.36%)
Benin	2017	5,354	2,608 (48.71%)	706 (48.06%)
Burkina faso	2021	5,018	1,432 (28.54%)	724 (29.18%)
Burundi	2017	4,902	1,827 (37.27%)	640 (40.25%)
Cameroon	2018	3,299	820 (24.86%)	739 (23.29%)
Cote d'ivoire	2021	3,850	1,680 (43.64%)	1,225 (40.03%)
DRC	2013	6,457	2,511 (38.89%)	3,405 (32.89%)
Gabon	2020	3,140	532 (16.94%)	22 (9.18%)
Gambia	2020	3,096	10 (0.32%)	1 (0.41%)
Ghana	2022	3,480	677 (19.45%)	403 (15.27%)
Madagascar	2021	4,167	335 (8.04%)	207 (6.43%)
Mali	2021	3,735	599 (16.04%)	588 (19.15%)
Mauritania	2020	3,826	22 (0.58%)	4 (0.62%)
Mozambique	2018	2,901	790 (27.23%)	1,234 (32.02%)
Nigeria	2018	9,599	3,796 (39.55%)	9,466 (37.63%)
Rwanda	2020	2,864	82 (2.86%)	40 (2.86%)
Tanzania	2022	3,704	225 (6.07%)	536 (6.98%)
Togo	2014	2,645	1,209 (45.71%)	361 (44.54%)
Uganda	2016	3,407	1,111 (32.61%)	1,436 (29.22%)
**Total**	-	79,739	21,007 (26.34%)	22,285 (27.95%)

*Footnote 1. Weighted counts were calculated using de-normalized sampling weights rescaled to an overall mean of 1.

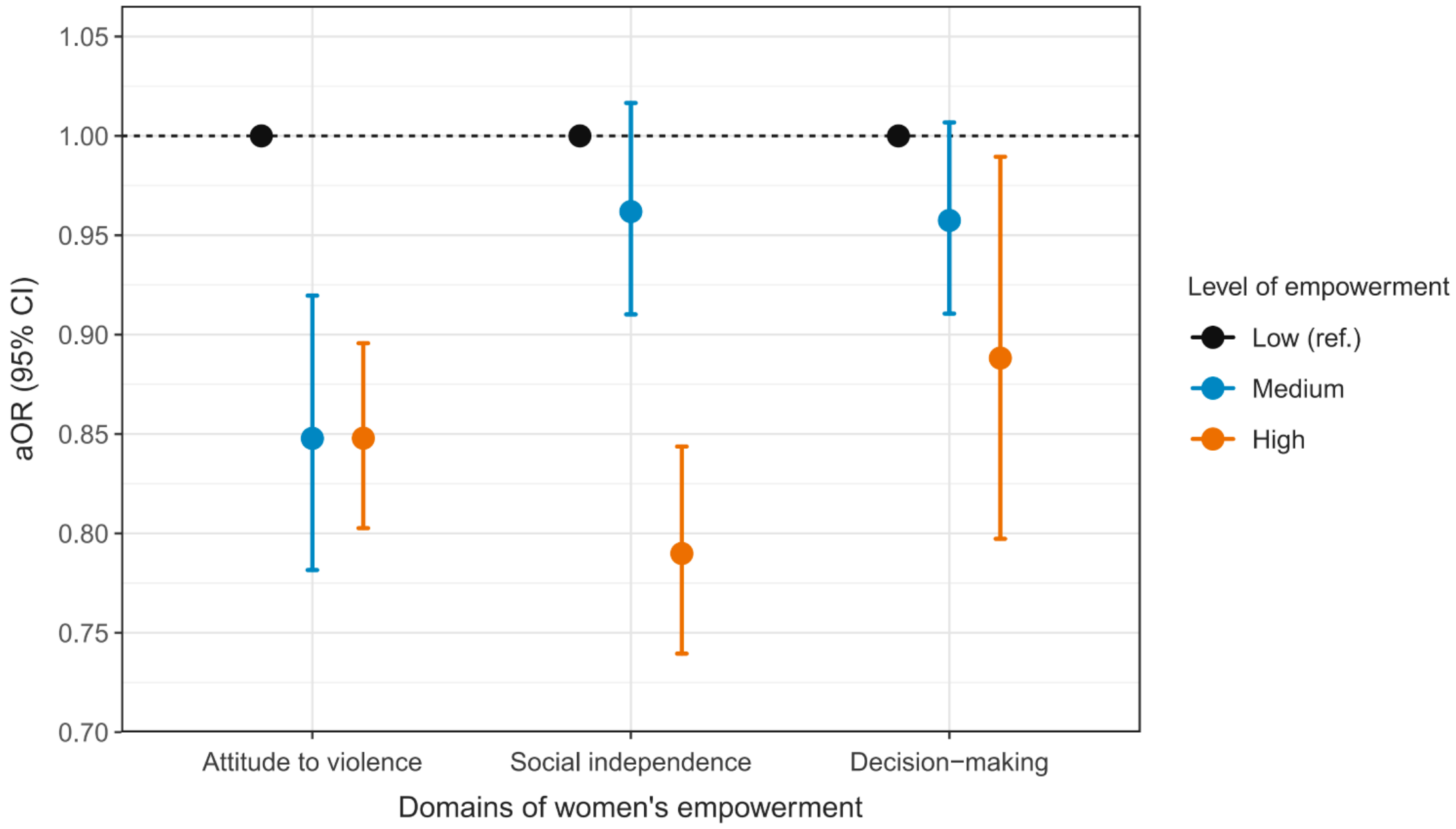

Figure S2. Forest plot of the association between women's empowerment and the childhood malaria positivity in 19 sub-Saharan African countries. aOR – adjusted odds ratio, CI – confidence interval.

Table S4. Sensitivity analysis of the main model

Exposure variables	Mean	SD	aOR[*] (95% CI)
Attitude to violence	-0.12	1.84	0.93 (0.90, 0.95) [†]
Social independence	-0.29	1.33	0.84 (0.82, 0.87) [†]
Decision-making	-1.21	1.07	0.95 (0.93, 0.97) [†]

aOR – adjusted odds ratio, SD – standard deviation.

[*]Footnote 1. The adjusted model included all three SWPER domains, maternal age, maternal employment, child age, child sex, birth order, birth interval, residence, household wealth index and country fixed effects, with a PSU random intercept. it represents the OR for each standard deviation increase in each SWPER domain.

[†]Footnote 2. P <0.001.

Table S5. Associations between women's empowerment and childhood malaria before and after additional adjustment for the child's bed net utilization

Domain	Category	Primary model, aOR[1] (95% CI)	Adjusted for child's bed net utilization, aOR (95% CI)
Attitude to violence	Low	Ref.	Ref.
	Medium	0.85 (0.78, 0.92)	0.85 (0.79, 0.92)
	High	0.85 (0.80, 0.90)	0.85 (0.80, 0.90)
Social independence	Low	Ref.	Ref.
	Medium	0.96 (0.91, 1.02)	0.96 (0.91, 1.02)
	High	0.79 (0.74, 0.84)	0.79 (0.74, 0.85)
Decision-making	Low	Ref.	Ref.
	Medium	0.96 (0.91, 1.01)	0.96 (0.91, 1.01)
	High	0.89 (0.80, 0.99)	0.89 (0.79, 0.99)

aOR – adjusted odds ratio, CI – confidence interval.

Table S6. Stratum-specific associations between women's empowerment and childhood malaria according to the child's bed net utilization

Domain	No bed net, aOR (95% CI)	Untreated bed net, aOR (95% CI)	ITN, aOR (95% CI)	Raw interaction *P*	BH-adjusted *P* (*q*)
Attitude to violence					
Medium (Ref. Low)	0.79 (0.70, 0.90)	1.95 (1.13, 3.36)	0.86 (0.77, 0.96)	0.011	0.017
High (Ref. Low)	0.86 (0.80, 0.93)	1.38 (0.93, 2.04)	0.83 (0.77, 0.89)	0.011	0.017
Social independence					
Medium (Ref. Low)	1.00 (0.92, 1.09)	1.14 (0.75, 1.74)	0.93 (0.87, 1.00)	0.625	0.625
High (Ref. Low)	0.79 (0.73, 0.87)	0.81 (0.53, 1.24)	0.79 (0.73, 0.86)	0.625	0.625
Decision-making					
Medium (Ref. Low)	0.91 (0.85, 0.98)	0.53 (0.37, 0.77)	1.01 (0.95, 1.08)	0.003	0.010
High (Ref. Low)	0.88 (0.75, 1.02)	0.67 (0.32, 1.41)	0.89 (0.77, 1.04)	0.003	0.010

The interaction *P* value represents the joint Wald test for all interaction terms within each SWPER domain and is therefore repeated across the two non-reference empowerment levels.
